# β-Lactolin, a Whey-Derived Lacto-Tetrapeptide, Prevents Alzheimer’s Disease Pathologies and Cognitive Decline

**DOI:** 10.3233/JAD-190997

**Published:** 2020-02-18

**Authors:** Yasuhisa Ano, Rena Ohya, Yuta Takaichi, Terukatsu Washinuma, Kazuyuki Uchida, Akihiko Takashima, Hiroyuki Nakayama

**Affiliations:** a Laboratory of Veterinary Pathology, Graduate School of Agricultural and Life Sciences, The University of Tokyo, Tokyo, Japan; b Research Laboratories for Health Science & Food Technologies, Kirin Holdings Company, Ltd., Kanagawa, Japan; c Faculty of Science, Gakushuin University, Tokyo, Japan

**Keywords:** Alzheimer’s disease, amyloid-β, β-lactolin, cognitive function, inflammation, memory, microglia, peptide, tauopathy

## Abstract

The prevention of age-related memory decline and dementia has been becoming a high priority because of the rapid growth in aging populations. Accumulating epidemiological and clinical studies indicate that intake of fermented dairy products rich in β-lactolin improves memory retrieval and executive function and attenuates cognitive decline in the elderly. However, the effects of long-term consumption of β-lactolin on Alzheimer’s disease (AD) pathologies have not been investigated. In the present study, we examined the effects of β-lactolin and whey digestion rich in β-lactolin on AD pathology in 5×FAD transgenic mice and PS19 tauopathy mice. Intake of β-lactolin and whey digestion rich in β-lactolin reduced the levels of inflammatory cytokines, suppressed the infiltration of activated microglia, decreased the levels of amyloid-β, ameliorated impaired long-term object memory, and attenuated decreased synaptophysin, dopamine, brain-derived neurotrophic factor, and insulin-like growth factor 1 levels in the cortex in 5×FAD transgenic mice. In addition, intake of β-lactolin and whey digestion rich in β-lactolin improved behavioral abnormality and reduced the ratio of phosphorylated tau to total tau in the cortex in PS19 tauopathy mice. These findings indicate that consumption with β-lactolin and whey digestion rich in β-lactolin suppresses inflammation and attenuates AD pathology and cognitive impairment.

## INTRODUCTION

With the rapid growth in the proportions of the older population worldwide, cognitive decline and dementia are becoming an increasing burden on not only patients and their families but also on national healthcare systems. Because of lack of a disease therapy for dementia, preventive approaches have been receiving increasing attention. Amyloid-β (Aβ) and phosphorylated tau become aggregated and are respectively deposited as senile plaques and neurofibrillary tangles (NFTs) in patients with Alzheimer’s disease (AD) [1, 2]. Accumulating evidence indicates that deposition of Aβ and phosphorylated tau induces inflammation in the brain and exacerbates neurological deficits and cognitive decline [3–5]. Inflammation in the brain is regulated by microglia, and microglia plays a key role in maintaining the neuroenvironment by removing pathogens, waste products, and old synapses via phagocytosis and by promoting synapse extension [6]. Proliferation and activation of microglia in the brain around senile plaques and NFTs are prominent features in AD [4], and activated microglia are known to be associated with disease progression. Therefore, regulating microglial function has been attracting increasing attention in the therapy and prevention of dementia, including AD [7, 8].

Accumulating evidence indicates that consumption of certain dairy products reduces the risk of cognitive decline in elderly individuals and AD patients. Crichton et al. revealed that individuals who consumed low-fat dairy products, including yogurt and cheese, once a week had higher cognitive function (memory recall) and social functioning than did those who did not [9]. Ozawa et al. investigated the dietary pattern and its potential association with reduced risk of dementia in more than 1000 dementia-free 60- to 79-year-old Japanese participants living in a local community [10, 11], and they found that inclusion of milk or fermented dairy products in the diet reduced the risk of dementia in the general Japanese population. In addition, our previous study demonstrated that intake of a dairy product (i.e., Camembert cheese) fermented with *Penicillium candidum* suppressed Aβ deposition and activation of microglia in the brain in an AD mouse model (5×FAD mice) [12]. These findings suggest that some ingredients, such as peptides generated during fermentation, suppress inflammation in the brain, attenuate cognitive decline, and promote healthy brain function during aging [13]. However, the underlying mechanism remains to be elucidated [14].

β-Lactolin is rich in Camembert cheese and other cheeses fermented by *Penicillium* [15]. β-Lactolin has been shown to increase monoamine levels in the frontal cortex and hippocampus and improve spatial working memory and attention in pharmacologically induced amnesia mice. Our previous studies showed that supplementation with β-lactolin-rich whey peptides improved memory retrieval, attention, and executive function in healthy adults [16, 17], and the findings suggest that consumption of β-lactolin-rich whey peptides is associated with activation of the frontal cortex, especially the dorsolateral prefrontal cortex regulating memory retrieval and executive function. However, the effects of β-lactolin on dementia and cognitive decline remain unclear. Particularly, the effects of long-term consumption of β-lactolin, the responsible ingredient in whey digestion, on dementia have not been investigated. Therefore, in the present study, we investigated the effects of β-lactolin on cognitive function and inflammation in AD model mice (5×FAD mice [amyloid model mice] and PS19 mice [tauopathy model mice]).

## MATERIALS AND METHODS

### Materials

**Fig.1:**
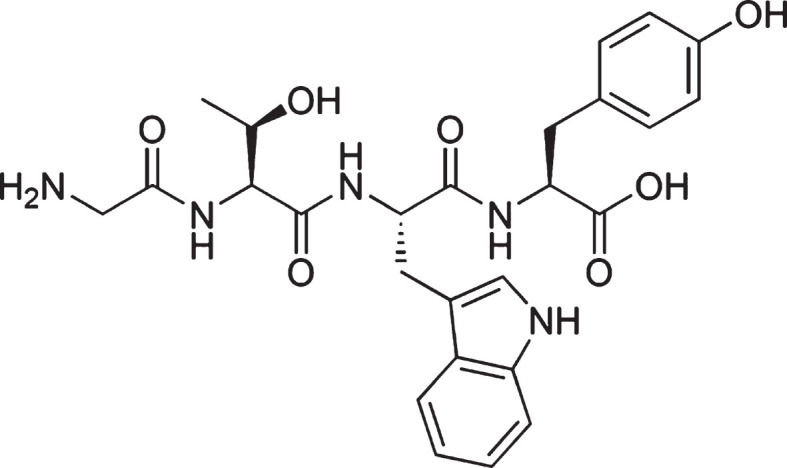
Chemical structure of β-lactolin. The chemical structure of β-lactolin, glycine-thereonine-tryptophan-tyrosine (GTWY) lactotetrapeptide.

GTWY peptide, β-lactolin, (purity: 98%; Fig. 1) was purchased from Bachem (Bubendorf, Switzerland). Whey digestion (containing 1.6 mg of β-lactolin per 1 g of whey digestion) was prepared by Kirin Holdings Company (Tokyo, Japan).

### Animals

AD model mice, B6SJL-Tg mice (APPSwFlLon, PSEN1*M146L*L286V, http://jaxmice.jax.org/strain/006554.html, [18]), hereafter referred to as 5×FAD transgenic mice, were purchased from Jackson Laboratory (Sacramento, CA, USA) and were maintained by crossing hemizygous transgenic mice with B6SJLF1/J mice in the experimental facility at the University of Tokyo. 5×FAD transgenic mice overexpress mutant human APP (695) with Swedish (K670N, M671L), Florida (I716V), and London (V717I) Familial Alzheimer’s Disease (FAD) mutations, along with human PS1 harboring 2 FAD mutations, M146L and L286V. Tauopathy model mice (B6;C3-Tg mice (Prnp-MAPT*P301S)PS19Vle/J, https://www.jax.org/strain/008169, [19]), hereafter referred to as PS19 mice, were also used in the present study. PS19 mice overexpress the T34 isoform of microtubule-associated protein tau with one N-terminal insert and four microtubule-binding repeats encoding the human P301S mutation. Nontransgenic wild-type littermates were used as controls in this study.

All experiments were approved by the Animal Care and Use Committee of the Graduate School of Agricultural and Life Sciences, the University of Tokyo, and they were conducted from May 2017 to June 2018 in strict accordance with their guidelines (Approval No; P15-042). All efforts were made to minimize animal suffering. Mice were maintained at room temperature (23°C±1°C) under constant 12-h light/dark cycles (light on from 8:00 am to 8:00 pm). Mice aged < 3 months were fed a standard purified rodent growth diet (AIN-93G; Oriental Yeast, Tokyo, Japan), and those aged≥3 months were fed a maintenance diet (AIN-93M; Oriental Yeast).

To evaluate the effects of β-lactolin and whey digestion rich in β-lactolin on Alzheimer-like disease, we fed 2.5-month-old transgenic 5×FAD and wild-type male mice with a diet that either contained or did not contain 0.05% w/w β-lactolin or 5% w/w whey digestion for 3.5 months (wild-type mice, *n* = 10; transgenic control mice, *n* = 10; transgenic mice with β-lactolin, *n* = 11; transgenic mice with whey digestion, *n* = 10). In addition, we also fed 3-month-old PS19 and wild-type male mice with a diet that either contained or did not contain 0.05% w/w β-lactolin or 5% w/w whey digestion for 6 months (wild-type mice, *n* = 12; transgenic control mice, *n* = 11; transgenic mice with β-lactolin, *n* = 11; transgenic mice with whey digestion, *n* = 11). The daily dosage of β-lactolin was 1.75 mg in the group fed with 0.05% w/w β-lactolin and 0.28 mg in the group fed with 5% w/w whey digestion when the consumption of food was 3.5 g. There were no significant differences in body weight and food consumption among different mouse groups (data not shown). After behavioral evaluations, mice were euthanized, and the brains were removed, as described in the following sections.

### Quantification of cytokine, synaptophysin, Aβ, and tau by enzyme-linked immunosorbent assay (ELISA)

Homogenate samples of the left hippocampus and the cortex were prepared as described in a previous study [20]. The hippocampus or cortex was homogenized in tris-buffer solution (TBS) containing a protease inhibitor cocktail (BioVision, CA, USA) using a multi-beads shocker (Yasui Kikai, Osaka, Japan). The supernatant (first) was collected after centrifugation at 50,000×g for 20 min, and the pellet was homogenized again in TBS containing 1% Triton X-100 (Wako, Osaka Japan), and the supernatant (second) was collected after centrifugation at 50,000×g for 20 min. The total protein concentration of each supernatant was measured using the BCA Protein Assay Kit (Thermo-Scientific, Yokohama, Japan). The first supernatant was used to quantify soluble Aβ_1-42_ (Wako), phosphorylated tau (pS199, ThermoFisher Scientific, Yokohama, Japan), total tau (ThermoFisher Scientific), synaptophysin (LSBio, Seattle, WA, USA), BDNF (Promega, Madison, WI, USA), and IGF-1 (R&D Systems, Minneapolis, MN, USA) by ELISA and cytokines and chemokines by a Bio-Plex assay system (Bio-Rad, Hercules, CA, USA). The second supernatant was used to quantify insoluble Aβ_1-42_ (Wako) by ELISA.

### Immunohistochemistry

The right-brain hemispheres (*n* = 10-11 in each group) were fixed in 10% formalin solution (Wako), paraffin-embedded, and cut into 5-*μ*m serial sections to evaluate Aβ deposition and infiltration of activated microglia and astrocytes. The studied brain regions included the hippocampus and cerebral cortex (bregma 2.30 mm posterior). After dewaxed and rehydrated, the sections for Aβ assay were treated in 98% formic acids, and those for ionized calcium-binding adaptor molecule 1 (Iba-1) and glial fibrillary acidic protein (GFAP) measurements were autoclaved at 121°C for 10 min in 0.2% citrate buffer (pH 6.0) for antigen retrieval. The sections were then incubated with a blocking solution (8% w/v skimmed milk) for 30 min after inactivation of endogenous peroxidase with 3% H_2_O_2_ (Wako) in methanol for 5 min. Subsequently, the sections were incubated overnight at room temperature with primary antibodies, including monoclonal anti-human Aβ_x-42_ antibodies (12F4, Millipore, Billerica, MA, USA), polyclonal anti-Iba-1 antibodies (Wako), or polyclonal anti-GFAP antibodies (Dako, Glostrup, Denmark). After the sections were incubated with horseradish peroxidase coupled goat anti-mouse or rabbit IgG antibodies (4*μ*g/ml, Nichirei, Tokyo, Japan) for 1 h at room temperature, they were visualized with 3,3’-diaminobenzidine (Wako) and counterstained in hematoxylin. The size of the positive region per area was measured using the Image J image analysis software (NIH, Bethesda, MD, USA).

### Assessment of microglial characteristics using a flow cytometer

For microglial activity evaluation, the right-brain hemispheres (*n* = 5 in each group) were removed, and the complement receptor (CD)11b-positive microglia were isolated and were used for flow cytometry analysis, as described in previous studies [12, 20, 21]. Brain cells were obtained by papain treatment using a Neural Tissue Dissociation Kit (P) (Miltenyi Biotec, MA, USA). The cells were treated with 2*μ*g/ml of anti-CD11b antibody conjugated with microbeads (Miltenyi Biotec), and CD11b-positive cells were isolated by magnetic cell sorting.

To measure the expression of cell surface markers, isolated microglia with more than 90% purity were stained with anti-CD11b-APC-Cy7 (M1/70, BD Pharmingen) and anti-CD86-PE (GL-1, eBioscience) and were then analyzed using a FACS Canto II flow cytometer (BD Bioscience).

To measure intracellular cytokine production, isolated microglia were plated in a 96-well plate (BD Biosciences, MA, USA) at 50,000 per well and were cultured in DMEM/F-12 (Gibco, CA, USA) medium supplemented with 10% fetal calf serum (Gibco) and 100 U/ml of penicillium/streptomycin (Sigma-Aldrich, MO, USA). Microglia were treated with a leukocyte activation cocktail using BD GolgiPlug (BD Biosciences) for 12 hours and were then fixed with a BD Cytofix/Cytoperm Fixation/Permeabilization kit (BD Biosciences), and subsequently, they were stained with following antibodies: anti-interleukin 1 beta (IL-1β)-FITC (NJTEN3, eBiosciences), anti-tumor necrosis factor (TNF)-*α*-APC (MP6-XT22, BD Pharmingen, CA, USA), and anti-CD11b-APC-Cy7 (M1/70, BD Pharmingen). The cells were analyzed using a flow cytometer.

### Dopamine analysis

To evaluate the level of dopamine in the brain, the tissue was homogenized in 0.2 M perchloric acid (PCA, Wako) containing 100*μ*M EDTA•2Na (Sigma-Aldrich). After centrifugation, the supernatant was analyzed by HPLC using an EICOMPAK SC-5ODS column and a PREPAK column (Eicom, Kyoto, Japan) with an electrochemical detection (ECD) unit. The mobile phase consisted of 83% 0.1 M acetic acid in a citric acid buffer (pH 3.5), 17% methanol (Wako), 190 mg/ml of sodium 1-octane sulfonate sodium (Wako), and 5 mg/ml EDTA•2Na. For ECD, the applied voltage was 750 mV via an Ag/AgCl reference electrode.

### Novel object recognition test

The novel object recognition test was performed during the light period in a polyvinyl chloride box (40×40×40 cm^3^) without a roof to evaluate the object memory. In the acquisition trial, a pair of wooden triangle poles (4.5×4.5×4.5 cm^3^) or wooden pyramids (4.5×4.5×4.5 cm^3^) was used; in the retention trial, a pair of poles or pyramids and a golf ball (4.5-cm diameter) were used. Mice were moved into the sound isolated room 16 h before the test started, and behavioral evaluations were carried out during the light phase. In all trials, the objects were placed 7.5 cm apart from the corner of the box. In the acquisition trial, each mouse was allowed to explore the box with the 2 objects for 10 min. Twenty-four hours after the acquisition trial, the mouse was allowed to explore the box with the novel and familiar objects for 5 min. The discrimination index was calculated by dividing the difference in the time for exploring the novel object and for the familiar object by the total time spent exploring both objects [(novel object exploration time – familiar object exploration time) / (total exploration time)]. A discrimination index of 0 indicated equal exploration of both objects.

### Open field test

To evaluate the activity in the novel place, the mice were subjected to the open field test in an open field box (40 cm×40 cm×40 cm, gray polyvinyl chloride) without a roof for 5 min. The mouse activity was monitored using the SMART video tracking software (PanLab Harvard Apparatus, MA, USA).

### Statistical analysis

Data are presented as mean±SEM. Data were analyzed using one-way ANOVA followed by the Tukey–Kramer, Dunnett, or Student’s *t*-tests, as described in figure legends. All statistical analyses were performed using the Ekuseru-Toukei 2012 software program (Social Survey Research Information, Tokyo, Japan).

## RESULTS

### Effects of β-lactolin on inflammation and microglial activation in 5×FAD mice

The levels of cytokines and chemokines in the cortex and hippocampus were measured to evaluate the effects of β-lactolin and whey peptides rich in β-lactolin on inflammation. The levels of IL-1β, macrophage inflammatory protein (MIP)-1*α*, MIP-1β, TNF-*α*, and IL-12p70 were reduced significantly in the cortex and hippocampus in the 5×FAD mice fed with β-lactolin compared with control 5×FAD mice (Table 1). In addition, the levels of IL-1*α*, IL-1β, MIP-1*α*, and IL-12p70 were reduced significantly in the cortex and hippocampus in 5×FAD mice fed with whey digestion compared with control 5×FAD mice (Table 1).

**Table 1:** Cytokine and chemokine levels in the frontal cortex

	Wild-Type	5×FAD		
	Ctrl	Ctrl	β-Lactolin	BL-W
IL-1*α*	0.74±0.15	1.29±0.13*	1.00±0.11	0.71±0.08^†^
IL-1β	0.35±0.06	0.49±0.02*	0.30±0.04^†^	0.39±0.03^†^
MIP-1*α*	0.30±0.03	15.08±1.56*	8.86±1.17^†^	9.01±1.34^†^
MIP-1β	7.62±1.06	9.75±0.94*	5.69±0.82^†^	6.16±0.50
TNF-*α*	5.30±0.61	7.62±0.64*	5.68±0.51^†^	5.30±0.81
IL-12p70	3.26±0.60	4.99±0.54**	2.68±0.36^††^	2.72±0.37^††^

Transgenic 5×FAD and wild-type male mice aged 2.5 months were fed a diet with or without 0.05% w/w β-lactolin or 5% w/w β-lactolin-rich whey enzymatic digestion (BL-W) for 3.5 months. The levels of cytokines and chemokines in the hippocampus were measured by ELISA. Data are represented as mean±SEM (sample size: wild-type mice, 10; control transgenic mice, 10; transgenic mice fed with β-lactolin, 11; or transgenic mice fed with BL-W, 10). *p*-values shown in the graph were calculated by one-way ANOVA followed by the Tukey–Kramer test. *and ^†^*p* < 0.05 and **and ^††^*p* < 0.01.

**Fig.2:**
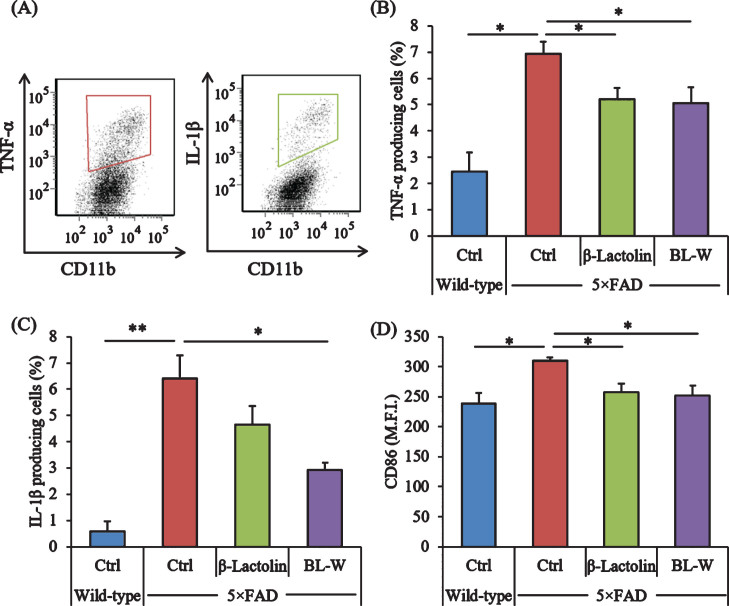
Effects of β-lactolin on inflammation and microglial activation in 5×FAD mice. Transgenic 5×FAD and wild-type male mice aged 2.5 months were fed a diet with or without 0.05% w/w β-lactolin or 5% w/w β-lactolin-rich whey enzymatic digestion (BL-W) for 3.5 months. A) Characterization of CD11b-positive microglia producing TNF-*α* and IL-1β by flow cytometry. B, C) The percentage of TNF-*α* or IL-1β-producing cells to CD11b-positive cells. D) Expressions of CD86 in CD11b-positive cells. M.F.I. is the mean fluorescent intensity. Data are presented as mean±SEM values (5 mice per group). *p*-values shown in the graph were calculated by one-way ANOVA followed by the Tukey–Kramer test. *p < 0.05, **p < 0.01.

Microglia in the brain were characterized using a flow cytometer to evaluate the effects of β-lactolin and whey digestion rich in β-lactolin on microglial activation. The ratios of TNF-*α* and IL-1β-producing cells to CD11b-positive cells (Fig. 2A) were higher in control 5×FAD mice than in wild-type mice (Fig. 2A) but were lower in 5×FAD mice fed with β-lactolin and whey digestion than in control 5×FAD mice (Fig. 2B, C, respectively). The expressions of CD86 in CD11b-positive microglia were significantly higher in control 5×FAD mice than in wild-type mice but were significantly lower in 5×FAD mice fed with β-lactolin and whey digestion than in control 5×FAD mice (Fig. 2D).

**Fig.3:**
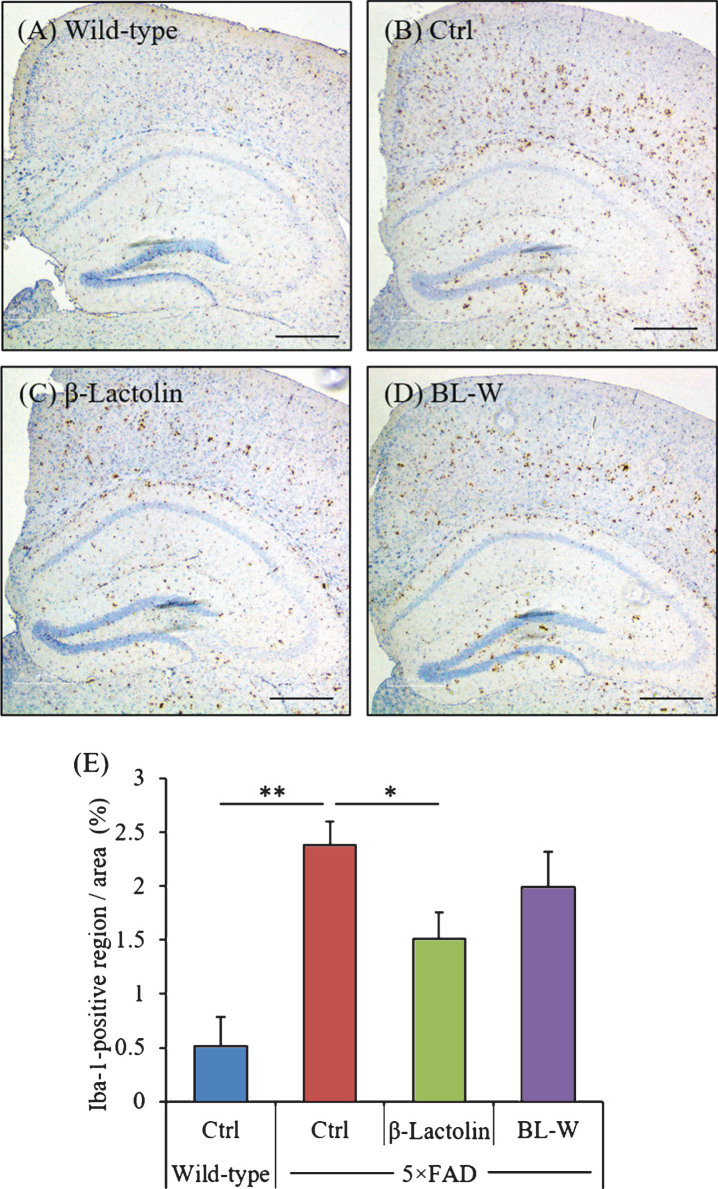
Effects of β-lactolin on microglial infiltration in 5×FAD mice. Transgenic 5×FAD and wild-type male mice aged 2.5 months were fed a diet with or without 0.05% w/w β-lactolin or 5% w/w β-lactolin-rich whey enzymatic digestion (BL-W) for 3.5 months. A-D) Representative immunohistochemistry images for Iba-1 in wild-type mice, transgenic control mice (Ctrl), and transgenic mice fed a diet containing β-lactolin or BL-W. Scale bars, 400*μ*m. E) Percentage of the Iba-1-positive area detected by immunohistochemistry in the cortex in transgenic control mice (Ctrl) and transgenic mice fed a diet containing β-lactolin or BL-W. Data are presented as means±SEM (sample size: wild-type mice, 10; control transgenic mice, 10; transgenic mice fed with β-lactolin, 11; or transgenic mice fed with BL-W, 10). *p*-values shown in the graph were calculated by one-way ANOVA followed by the Tukey–Kramer test. *p < 0.05.

The distribution of activated microglia in the brain was observed using immunohistochemistry (Fig. 3A-D). The levels of Iba-1-positive microglia in the cortex were reduced significantly in 5×FAD mice fed with β-lactolin compared with control 5×FAD mice (Fig. 3E). The distribution of activated astrocytes in the brain was also observed using immunohistochemistry (Supplementary Figure 1A-D). The levels of GFAP-positive astrocytes in the cortex were reduced in 5×FAD mice fed with β-lactolin compared with control 5×FAD mice (Supplementary Figure 1E).

These findings indicate that consumption of β-lactolin and whey digestion rich in β-lactolin suppresses inflammation and microglial activation in 5×FAD mice.

### Effects of β-lactolin on Aβ deposition in 5×FAD mice

**Fig.4:**
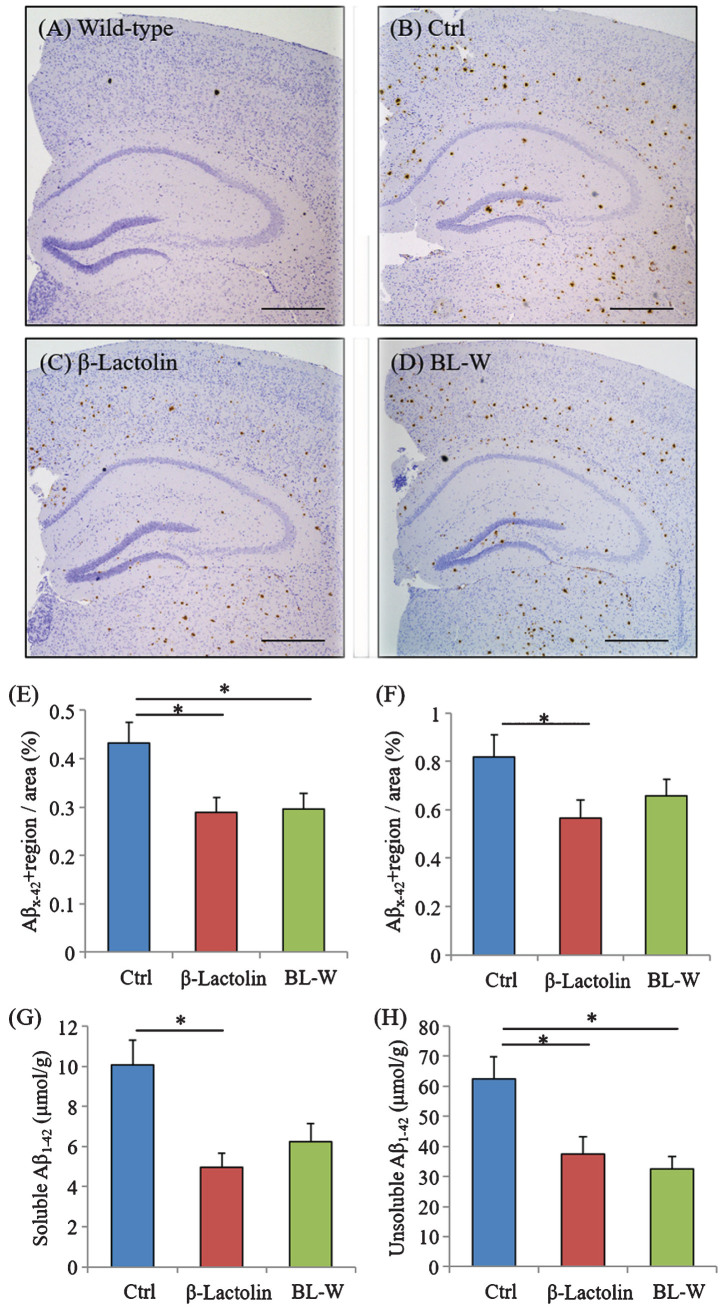
Effects of β-lactolin on Aβ deposition in 5×FAD mice. Transgenic 5×FAD and wild-type male mice aged 2.5 months were fed a diet with or without 0.05% w/w β-lactolin or 5% w/w β-lactolin-rich whey enzymatic digestion (BL-W) for 3.5 months. A-D) Representative immunohistochemistry images for Aβ_1-42_ in wild-type mice and transgenic mice with or without β-lactolin or β-lactolin-rich whey enzymatic digestion. Scale bars, 400*μ*m. E, F) Percentage of the Aβ_1-42_-positive area detected by immunohistochemistry in the cortex and hippocampus in transgenic control mice (Ctrl) and transgenic mice fed a diet containing β-lactolin or BL-W. G, H) The levels of TBS-soluble or TBS-insoluble/TBS-T soluble Aβ_1-42_ in the frontal cortex. Data are presented as means±SEM (sample size: wild-type mice, 10; control transgenic mice, 10; transgenic mice fed with β-lactolin, 11; or transgenic mice fed with BL-W, 10). *p*-values shown in the graph were calculated by one-way ANOVA followed by the Tukey–Kramer test. *p < 0.05.

5×FAD mice were fed with β-lactolin and whey digestion, and Aβ levels were measured using immunohistochemistry and ELISA to assess the effects of β-lactolin and whey digestion rich in β-lactolin on Aβ deposition in the brain. Immunohistochemical measurements (Fig. 4A-D) showed that Aβ_1-42_ levels in the somatosensory/visual cortex were significantly lower in 5×FAD mice fed with β-lactolin and whey digestion than in control 5×FAD mice (Fig. 4E). Aβ_1-42_ levels in the hippocampus were significantly lower in 5×FAD mice fed with β-lactolin, but not in those with whey digestion, than in control 5×FAD mice (Fig. 4F).

Quantification by ELISA revealed that the levels of TBS-soluble Aβ_1-42_ and TBS-insoluble and TBS-T soluble Aβ_1-42_ were significantly lower in the cortex in 5×FAD mice fed with β-lactolin and whey digestion than in control 5×FAD mice (Fig. 4G, H). These results indicate that the consumption of β-lactolin and whey peptides rich in β-lactolin reduces the deposition of Aβ_1-42_, especially in the cortex.

### Effects of β-lactolin on memory impairment in 5×FAD mice

**Fig. 5:**
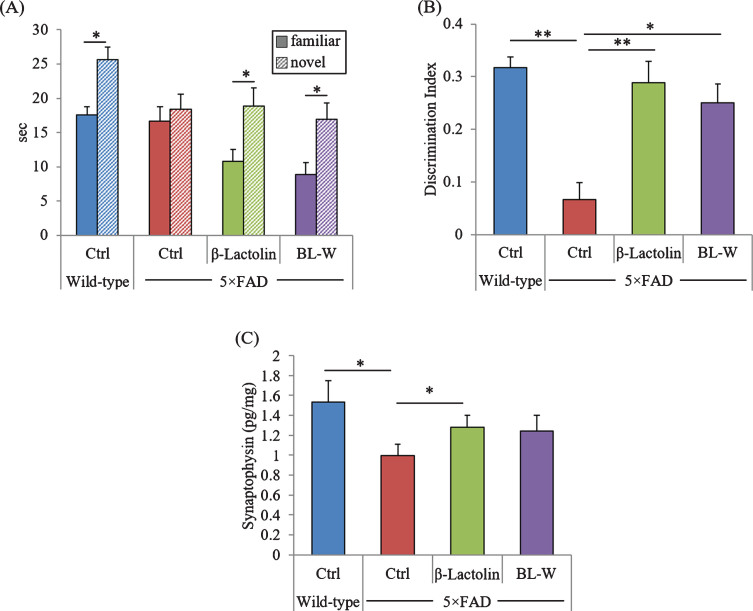
Effects of β-lactolin on memory function in 5×FAD mice. Transgenic 5×FAD and wild-type male mice aged 2.5 months were fed a diet with or without 0.05% w/w β-lactolin or 5% w/w β-lactolin-rich whey enzymatic digestion (BL-W) for 3.5 months. Mice aged 6 months were subjected to the novel object recognition test to evaluate object recognition memory. A, B) The time spent exploring novel and familiar objects during 5 min of re-exploration (A) and the discrimination index [(time spent with object A – time spent with object B) / total time exploring both objects] (B) were measured. C) The levels of synaptophysin in the frontal cortex. Data are presented as means±SEM (sample size: wild-type mice, 10; control transgenic mice, 10; transgenic mice fed with β-lactolin, 11; or transgenic mice fed with BL-W, 10). *p*-values shown in the graph were calculated by student *t*-test or one-way ANOVA followed by the Tukey–Kramer test. *p < 0.05, **p < 0.01.

5×FAD mice underwent a novel object recognition test to evaluate object memory retrieval. The time approaching the novel object was significantly longer than that approaching the familiar object in wild-type mice and 5×FAD mice fed with β-lactolin and whey digestion, but there was no difference in the time approaching two objects in control 5×FAD mice (Fig. 5A). In addition, the discrimination indexes were reduced significantly in control 5×FAD mice compared with wild-type mice but were increased significantly in 5×FAD mice fed with β-lactolin and whey digestion compared with control 5×FAD mice (Fig. 5B). These results indicate that consumption of β-lactolin and whey digestion ameliorates long-term object memory impairment in 5×FAD mice.

The levels of synaptophysin in the frontal cortex were measured to evaluate the effects of β-lactolin and whey peptides rich in β-lactolin on synapse. The levels of synaptophysin were reduced significantly in 5×FAD mice compared with wild-type mice (Fig. 5C) but were significantly increased in 5×FAD mice fed with β-lactolin (Fig. 5C) compared with control 5×FAD mice. In addition, synaptophysin levels also increased in 5×FAD mice fed with whey digestion compared with control 5×FAD mice (Fig. 5C), although the difference was not significant. The results indicate that β-lactolin and whey peptides rich in β-lactolin suppress synapse loss in 5×FAD mice.

### Effects of β-lactolin on dopamine, brain-derived neurotrophic factor (BDNF), and insulin-like growth factor 1 (IGF-1) levels

To evaluate the effects of β-lactolin and whey peptides rich in β-lactolin on neuroprotection, the levels of dopamine, BDNF, and IGF-1 in the cortex were measured. The levels of dopamine, BDNF, and IGF-1 were reduced significantly in 5×FAD mice compared with wild-type mice (Supplementary Figure 2A-C). The levels of dopamine and BDNF were significantly increased in 5×FAD mice fed with β-lactolin compared with control 5×FAD mice (Supplementary Figure 2A, B). In addition, IGF-1 levels in 5×FAD mice fed with β-lactolin or whey digestion and BDNF levels in 5×FAD mice fed with whey digestion also increased compared with those in control 5×FAD mice, although the differences were not significant (Supplementary Figure 2A-C). These results indicate that β-lactolin and whey peptides rich in β-lactolin increase BDNF and IGF-1 levels in 5×FAD mice.

### Effects of β-lactolin on behavioral abnormalities and tau levels in PS19 mice

**Fig.6:**
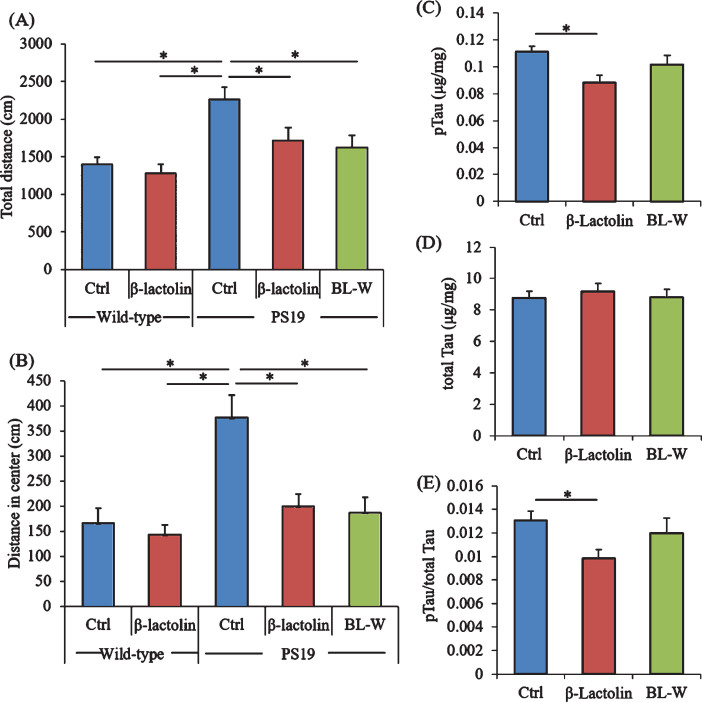
Effects of β-lactolin on memory function and tau accumulation in PS19 mice. Transgenic PS19 and wild-type male mice aged 3 months were fed a diet with or without 0.05% w/w β-lactolin or 5% w/w β-lactolin-rich whey enzymatic digestion (BL-W) for 6 months. Mice aged 9 months were subjected to the open field test to evaluate behavioral abnormality. A, B) The total distances in the open field (A) and in the center of the open field (B). C, D) The levels of phosphorylated tau (pTau, C) and total tau (D) in the cortex. E) The ratio of pTau to total tau. Data are presented as means±SEM (sample size: wild-type mice, 12; control transgenic mice, 11; transgenic mice fed with β-lactolin, 11; or transgenic mice fed with BL-W, 11). *p*-values shown in the graph were calculated by one-way ANOVA followed by the Tukey–Kramer test. *p < 0.05.

PS19 mice underwent the open field test to evaluate activity and anxiety. The total distances in the open field box were significantly increased in control PS19 mice compared with wild-type mice but were significantly reduced in PS19 mice fed with β-lactolin and whey digestion compared with control PS19 mice (Fig. 6A). The distances in the center zone of the open field box were significantly increased in control PS19 mice compared with wild-type mice but were reduced in PS19 mice fed with β-lactolin and whey digestion compared with control PS19 mice (Fig. 6B).

The levels of pTau and total tau in the cortex were measured to evaluate the effects of β-lactolin and whey peptides rich in β-lactolin on tau pathologies. The levels of TBS-soluble pTau were reduced significantly in PS19 mice fed with β-lactolin compared with control PS19 mice (Fig. 6C), but the levels of total tau were not changed (Fig. 6D). The ratio of pTau to total tau was significantly reduced in the PS19 mice fed with β-lactolin compared with control PS19 mice (Fig. 6E). In addition, synaptophysin and IGF-1 levels in PS19 mice fed with β-lactolin and BDNF levels in PS19 mice fed with whey digestion increased compared with those in control PS19 mice (Supplementary Figure 5A-C). The results indicate that β-lactolin and whey peptides rich in β-lactolin suppress cognitive impairment and tau accumulation in PS19 mice.

## DISCUSSION

Accumulating studies indicate that fermented dairy products rich in β-lactolin improve cognitive performance and dementia [15, 17]. However, the effects of β-lactolin on AD pathologies and cognitive decline have not been investigated. This study found that long-term intakes of β-lactolin and whey digestion rich in β-lactolin suppressed microglial activation, inflammation, and Aβ deposition in the cortex and attenuated memory impairments and synapse loss in 5×FAD mice. In addition, the levels of pTau in the cortex were also suppressed in PS19 mice. Notably, β-Lactolin also attenuated the reduction of dopamine, BDNF, and IGF-1 in the cortex in 5×FAD and PS19 mice. The present study suggests that β-lactolin contributes to the ameliorating effects of dairy products on dementia as demonstrated by epidemiological studies.

Accumulating evidence [16, 17] indicates that supplementation with whey digestion rich in β-lactolin improves memory retrieval and attention. However, the effects of β-lactolin on brain inflammation in AD have not been investigated. We found that long-term intakes of β-lactolin and whey digestion rich in β-lactolin suppressed the infiltration of activated microglia and decreased the production of IL-1β and TNF-*α* and the expression of CD86 in microglia in the brain. Inflammatory-type M1 microglia are characterized by the production of IL-1β and TNF-*α* and the expression of CD86 [22, 23]; therefore, β-lactolin might suppress the inflammatory-type microglial activation. Studies have indicated that inflammatory-type M1 microglia promote synapse loss and memory impairment, thereby exacerbating the pathologies in AD [4, 8]; accordingly, recent studies have targeted activated microglia as a therapeutic target in AD [24]. Studies have demonstrated that lipopolysaccharide inoculation in the brain increases the levels of cytokines and chemokines, including IL-1β and TNF-*α*, in the brain and impairs the memory function [25], and iso-*α*-acids suppress the activation of microglia, prevent inflammation and Aβ deposition, and improve memory impairment in 5×FAD mice [20]. Moreover, inflammation is associated with the synapse loss [26, 27], and increased IL-1β reduces the levels of synaptophysin and suppresses axon development through activation of the p38-MAPK signaling pathway in the brain [28]. Notably, inflammation in the brain also suppresses the production of BDNF and IGF-1, which have neuroprotective effects, including improved synapse formation and memory retrieval [29]. Collectively, the effects of β-lactolin and whey digestion rich in β-lactolin on suppression of microglial activation might attenuate AD pathology and cognitive decline.

The present study indicates that β-lactolin and whey digestion rich in β-lactolin prevent cognitive impairment induced by not only Aβ but also tau in the model mice. Studies have demonstrated that 5×FAD exhibit object memory impairment, indicated by reduced time approaching the novel object, at 5 to 6 months of age, and suppression of inflammation improves the memory impairment. In addition, PS19 mice at 5, 7, and 9 months of age exhibit behavioral abnormality, indicated by increased total distance in the open field test [30], and anti-inflammatory agent (FK506) suppresses tau pathology [19]. Moreover, intracerebroventricular treatment with lipopolysaccharide exacerbates pTau pathology in tauopathy mice, suggesting that inflammation promotes the production of pTau in the brain [5]. Notably, increased inflammation induces synaptic disruption [31]. These findings suggest that anti-inflammatory effects of β-lactolin might contribute to the attenuation of cognitive impairment and phosphorylation of tau.

Our previous studies have demonstrated that β-lactolin orally administered can enter the brain and inhibit the activity of monoamine oxidase B (MAO-B), thereby resulting in increased dopamine levels in the brain in normal ICR mice [15, 21], and dopamine D1-like receptor is involved in the memory improvement induced by β-lactolin in scopolamine-induced amnesia model mice [15]. In the present study, the levels of dopamine in the frontal cortex increased in 5×FAD mice fed with the diet containing β-lactolin and whey digestion rich in β-lactolin. It has been demonstrated that cortical dopamine, but not hippocampus dopamine, is crucial for object recognition memory in AD model mice [32]; dopamine is associated with microglial activation and inflammation in the brain, and dopamine inhibits lipopolysaccharide (LPS)-induced inflammatory responses, especially nitric oxide production, in murine microglia cells [33]. In addition, dopamine controls systemic inflammation through inhibition of NLRP3 inflammasome [34], and dopamine D1 receptor signaling prevents neuronal inflammation via suppression of NLRP3 inflammation. In the present study, β-lactolin and whey digestion rich in β-lactolin decreased the levels of IL-1β and TNF-*α* in the cortex, as well as in CD11b-positive microglia. The findings suggest that the preventive effects of β-lactolin on AD pathology and cognitive impairment are associated with the suppression of microglial inflammatory responses. Although further studies are needed to investigate the underlying mechanisms by which β-lactolin suppresses the activation of microglia, our present findings suggest increased dopamine levels induced by β-lactolin in the cortex are associated with the suppression of inflammation in the brain in 5×FAD mice.

The current study has some limitations. First, we did not evaluate the direct effects of β-lactolin on microglial activation, Aβ production, and synapse loss. We preliminary evaluated whether treatment with β-lactolin inhibits LPS-induced inflammatory responses of primary microglia *in vitro* and found that treatment with β-lactolin did not suppress TNF-*α* production enough (data not shown). This result suggests that β-lactolin does not directly suppress the activation of microglia. We speculated that MAO-B-inhibiting activity of β-lactolin increases the level of dopamine, thereby resulting in the suppression of inflammation and Aβ production; however, further studies are needed to confirm the effects of β-lactolin on microglia activation and Aβ production *in vitro* systems. Second, control wild type mice fed with β-lactolin and whey digestions were not included in this study.

Dementia is a serious social issue because of the increasing patient number, and no effective therapeutic approaches are available for this disease. Therefore, preventive approaches in daily life are attracting increasing attention. Nutritional ingredients have excellent safety profiles without long term side effects. β-Lactolin, which is derived from whey protein and is safer and easier to intake in daily life, might improve cognitive function and synapse reduction associated with Aβ deposition and tau phosphorylation.

## DISCLOSURE STATEMENT

Authors’ disclosures available online (https://www.j-alz.com/manuscript-disclosures/19-0997r1).

## Supplementary Material

Supplementary Figures
