# Supplementary material for: β-Lactolin, a Whey-Derived Lacto-Tetrapeptide, Prevents Alzheimer’s Disease Pathologies and Cognitive Decline
Source: J Alzheimers Dis. 2020 Feb 18;73(4):1331–42. doi: 10.3233/JAD-190997 (PMC7081097; doi:10.3233/JAD-190997)

**Supplementary Material**

**β-Lactolin, a Whey-Derived Lacto-Tetrapeptide, Prevents Alzheimer’s Disease Pathologies and Cognitive Decline**

**Supplementary Figure 1. Effects of β-lactolin on** **infiltration of astrocytes in 5×FAD mice.**

Transgenic 5×FAD and wild-type male mice aged 2.5 months were fed a diet with or without 0.05% w/w β-lactolin or 5% w/w β-lactolin-rich whey enzymatic digestion (BL-W) for 3.5 months. A-D) Representative immunohistochemistry images for GFAP in wild-type mice, transgenic control mice (Ctrl), and transgenic mice fed a diet containing β-lactolin or BL-W. Scale bars, 400 μm. E) Percentage of the GFAP positive area detected by immunohistochemistry in the frontal cortex in transgenic control mice (Ctrl) and transgenic mice fed a diet containing β-lactolin or BL-W. Data are presented as means ± SEM (sample size: wild-type mice, 10; control transgenic mice, 10; transgenic mice fed with β-lactolin, 11; or transgenic mice fed with BL-W, 10). ***p* < 0.01.


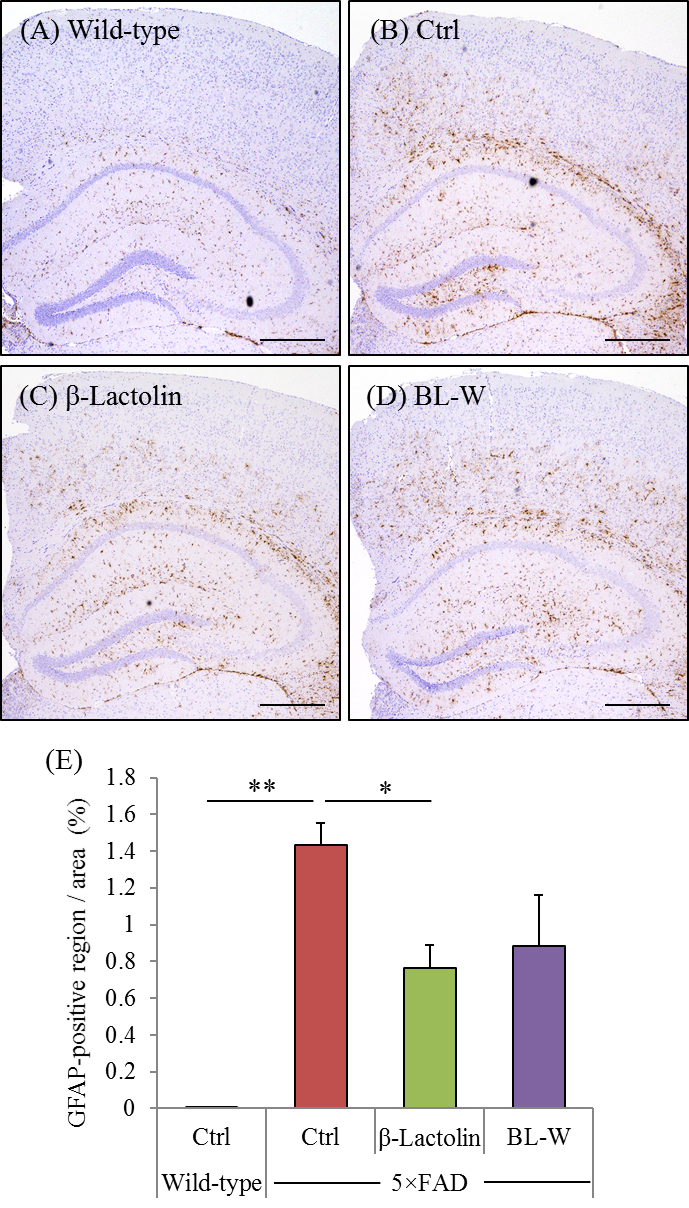


**Supplementary Figure 2. Effects of β-lactolin on the levels of dopamine and neurotrophic factors in 5×FAD mice.** Transgenic 5×FAD and wild-type male mice aged 2.5 months were fed a diet with or without 0.05% w/w β-lactolin or 5% w/w β-lactolin-rich whey enzymatic digestion (BL-W) for 3.5 months. A-C) The levels of dopamine, BDNF, and IGF-1 in the cortex. Data are presented as means ± SEM (sample size: wild-type mice, 10; control transgenic mice, 10; transgenic mice fed with β-lactolin, 11; or transgenic mice fed with BL-W, 10). p-values shown in the graph were calculated by one-way ANOVA followed by the Tukey–Kramer test. **p* < 0.05.


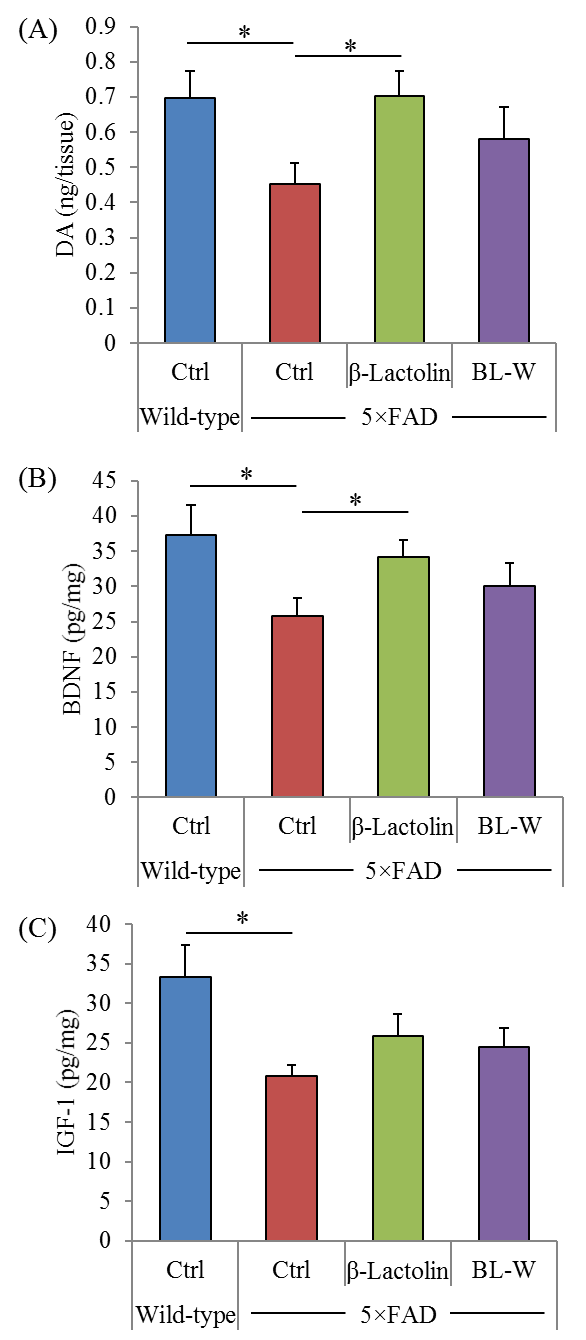


**Supplementary Figure 3. Effects of β-lactolin on the levels of synaptophysin and neurotrophic factors in PS19 mice.** Transgenic PS19 and wild-type male mice aged 3 months were fed a diet with or without 0.05% w/w β-lactolin or 5% w/w β-lactolin-rich whey enzymatic digestion (BL-W) for 6 months. A-C) The levels of synaptophysin, BDNF, and IGF-1 in the cortex. Data are presented as means ± SEM (sample size: wild-type mice, 12; control transgenic mice, 11; transgenic mice fed with β-lactolin, 11; or transgenic mice fed with BL-W, 11). p-values shown in the graph were calculated by one-way ANOVA followed by the Tukey–Kramer test. **p* < 0.05.


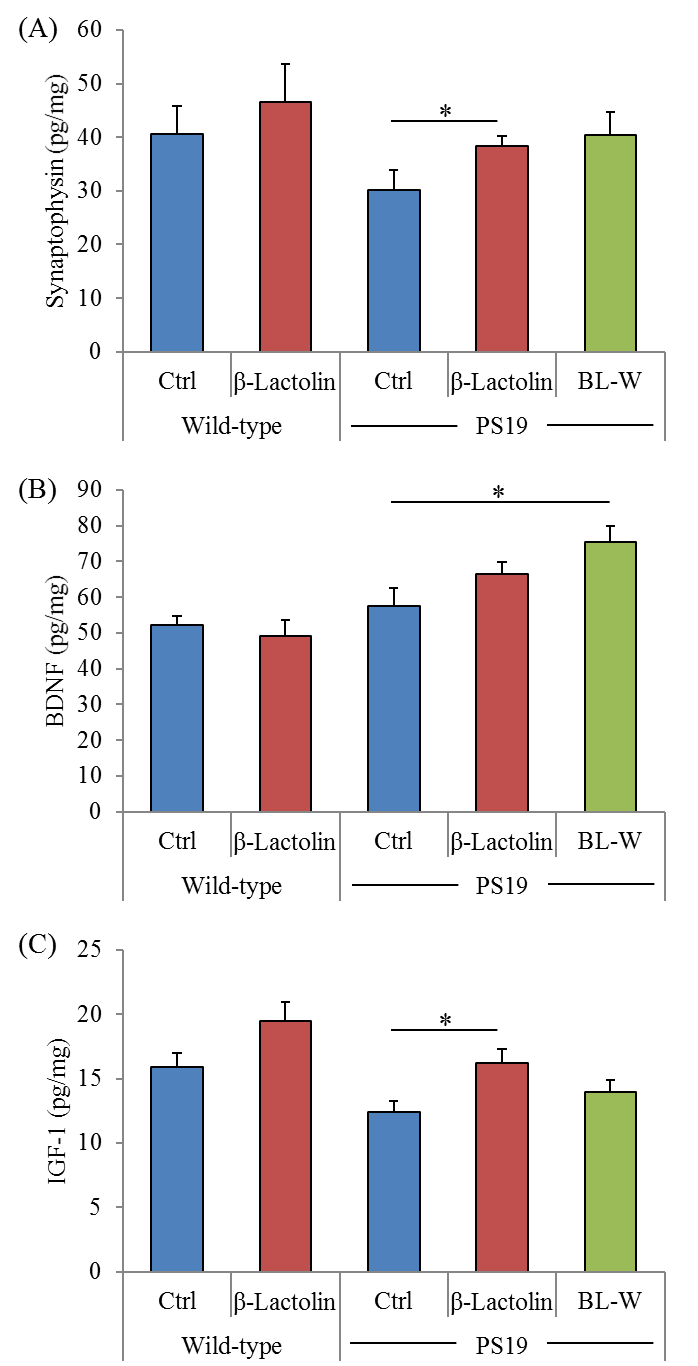

Supplement: Supplementary Figures [file jad-73-jad190997-s001.docx]
